# Supplementary material for: High Diversity of Giardia duodenalis Assemblages and Sub-Assemblages in Asymptomatic School Children in Ibadan, Nigeria
Source: Trop Med Infect Dis. 2023 Feb 28;8(3):152. doi: 10.3390/tropicalmed8030152 (PMC10051407; doi:10.3390/tropicalmed8030152)
Supplement: Supplementary file 1 [file tropicalmed-08-00152-s001.zip › Table S1 Tijani et al. TMID_2022.docx]

**Table S1.** Oligonucleotides used for the molecular identification and characterization of *Giardia duodenalis* in the present study.

| **Locus** | **Oligonucleotide** | **Sequence (5´–3´)** | **Reference** |
| --- | --- | --- | --- |
| *ssu* rRNA | Probe | FAM–CCCGCGGCGGTCCCTGCTAG–BHQ1 | [52] |
|  | Gd-80F | GACGGCTCAGGACAACGGTT | [52] |
|  | Gd-127R | TTGCCAGCGGTGTCCG | [52] |
| *ssu* rRNA | Gia2029 | AAGTGTGGTGCAGACGGACTC | [53] |
|  | Gia2150c | CTGCTGCCGTCCTTGGATGT | [53] |
|  | RH11 | CATCCGGTCGATCCTGCC | [54] |
|  | RH4 | AGTCGAACCCTGATTCTCCGCCAGG | [54] |
| *gdh* | GDHeF | TCAACGTYAAYCGYGGYTTCCGT | [55] |
|  | GDHiF | CAGTACACCTCYGCTCTCGG | [55] |
|  | GDHiR | GTTRTCCTTGCACATCTCC | [55] |
| *bg* | G7_F | AAGCCCGACGACCTCACCCGCAGTGC | [56] |
|  | G759_R | GAGGCCGCCCTGGATCTTCGAGACGAC | [56] |
|  | G99_F | GAACGAACGAGATCGAGGTCCG | [56] |
|  | G609_R | CTCGACGAGCTTCGTGTT | [56] |
| *tpi* | AL3543 | AAATIATGCCTGCTCGTCG | [57] |
|  | AL3546 | CAAACCTTITCCGCAAACC | [57] |
|  | AL3544 | CCCTTCATCGGIGGTAACTT | [57] |
|  | AL3545 | GTGGCCACCACICCCGTGCC | [57] |

*bg*: β-giardin; *gdh*: Glutamate dehydrogenase; *ssu* rRNA: Small subunit ribosomal RNA; *tpi*: Triose phosphate isomerase.
