# Supplementary material for: High Diversity of Giardia duodenalis Assemblages and Sub-Assemblages in Asymptomatic School Children in Ibadan, Nigeria
Source: Trop Med Infect Dis. 2023 Feb 28;8(3):152. doi: 10.3390/tropicalmed8030152 (PMC10051407; doi:10.3390/tropicalmed8030152)
Supplement: Supplementary file 1 [file tropicalmed-08-00152-s001.zip › Table S2 Tijani et al. TMID_2022.docx]

**Table S2.** PCR cycling conditions used for the molecular identification and/or characterization of the microeukaryotic parasites investigated in the present study.

|  | **Temperature and time** | | | |  |  |  |
| --- | --- | --- | --- | --- | --- | --- | --- |
| **Locus** | **Initial denaturation** | **Denaturation** | **Annealing** | **Extension** | **No. cycles** | **Final extension** | **Reference** |
| *ssu* rRNA | 95 °C 15 min | 95 °C 15 s | 60 °C 1 min | 72 °C 30 s | 45 | – | [52] |
| *ssu* rRNA | 95 °C 2 min | 95 °C 45 s | 58/55 °C 30 s | 72 °C 45 s | 35 | 72 °C 4 min | [53] |
| *gdh* | 95 °C 3 min | 95 °C 30 s | 55 °C 30 s | 72 °C 1 min | 35 | 72 °C 7 min | [55] |
| *bg* | 95 °C 7 min | 95 °C 30 s | 65/55 °C 30 s | 72 °C 1 min | 35 | 72 °C 7 min | [56] |
| *tpi* | 94 °C 5 min | 94 °C 45 s | 50 °C 45 s | 72 °C 1 min | 35 | 72 °C 10 min | [57] |

*bg*: β-giardin; *gdh*: Glutamate dehydrogenase; *ssu* rRNA: Small subunit ribosomal RNA; *tpi*: Triose phosphate isomerase.
