# Supplementary material for: High Diversity of Giardia duodenalis Assemblages and Sub-Assemblages in Asymptomatic School Children in Ibadan, Nigeria
Source: Trop Med Infect Dis. 2023 Feb 28;8(3):152. doi: 10.3390/tropicalmed8030152 (PMC10051407; doi:10.3390/tropicalmed8030152)
Supplement: Supplementary file 1 [file tropicalmed-08-00152-s001.zip › Table S4 Tijani et al TMID_2022.docx]

**Table S4.** Frequency and molecular diversity of *G. duodenalis* identified at the *ssu* locus in the schoolchildren population investigated in the present study. GenBank accession numbers are provided.

| **Assemblage** | **Sub-assemblage** | **No. isolates** | **Reference sequence** | **Stretch** | **Single nucleotide polymorphisms** | **GenBank ID** |
| --- | --- | --- | --- | --- | --- | --- |
| A | Unknown | 16 | AF199446 | 1–292 | None | OP946920 |
|  |  | 1 | AF199446 | 4–289 | C116Y | OP946921 |
| B | Unknown | 35 | AF199447 | 1–293 | None | OP946922 |
|  |  | 1 | AF199447 | 1–289 | C34Y, G112R | OP946923 |
|  |  | 1 | AF199447 | 4–290 | C101Y | OP946924 |
|  |  | 1 | AF199447 | 12–291 | C105Y | OP946925 |
|  |  | 1 | AF199447 | 24–293 | A130R | OP946926 |
|  |  | 1 | AF199447 | 1–290 | G154R | OP946927 |
|  |  | 1 | AF199447 | 16–293 | A166W | OP946928 |
