# Supplementary material for: High Diversity of Giardia duodenalis Assemblages and Sub-Assemblages in Asymptomatic School Children in Ibadan, Nigeria
Source: Trop Med Infect Dis. 2023 Feb 28;8(3):152. doi: 10.3390/tropicalmed8030152 (PMC10051407; doi:10.3390/tropicalmed8030152)
Supplement: Supplementary file 1 [file tropicalmed-08-00152-s001.zip › Table S5 Tijani et al TMID_2022.docx]

**Table S5.** Frequency and molecular diversity of *G. duodenalis* identified at the *gdh* locus in the schoolchildren population investigated in the present study. GenBank accession numbers are provided.

| **Assemblage** | **Sub-assemblage** | **No. isolates** | **Reference sequence** | **Stretch** | **Single nucleotide polymorphisms** | **GenBank ID** |
| --- | --- | --- | --- | --- | --- | --- |
| A | AII | 8 | L40510 | 64–491 | None | OP947099 |
|  |  | 1 | L40510 | 64–491 | T456Y | OP947100 |
| B | BIII | 1 | AF069059 | 40–388 | C87Y, C99Y, T138Y, T147Y, G150R, G189R, C309Y, C336Y | OP947101 |
|  |  | 1 | AF069059 | 40–455 | C87T, T147C, G150A, C29TC, G372A, C375T | OP947102 |
|  |  | 1 | AF069059 | 40–411 | C87T, C168T, C309T | OP947103 |
|  |  | 1 | AF069059 | 40–460 | C99T, C123T, T147C, G150A, C309T | OP947104 |
|  |  | 1 | AF069059 | 76–460 | C99Y, T147Y, G150R, C309T, G402R, T426Y, T456Y | OP947105 |
|  |  | 1 | AF069059 | 49–458 | C99T, T237C, G354A | OP947106 |
|  |  | 1 | AF069059 | 40–455 | C99T, C309T | OP947107 |
|  |  | 1 | AF069059 | 40–451 | C132T, T276C, C309T, C336T | OP947108 |
|  |  | 1 | AF069059 | 40–455 | T147C, C309T | OP947109 |
|  |  | 1 | AF069059 | 40–407 | C309T, C330T | OP947110 |
|  | BIV | 1 | L40508 | 76–496 | T183C, T366C, T387C, C432T | OP947111 |
|  |  | 1 | L40508 | 76–496 | A322G | OP947112 |
|  | BIII/BIV | 1 | L40508 | 116–491 | C123Y, T135Y, T183C, C255Y, C273Y, T309Y, C345Y, T366C, T387C, A438R | OP947113 |
|  |  | 1 | L40508 | 76–495 | T135Y, T183Y, G186R, C255Y, C258Y, C273Y, C345Y, T366Y, C372Y, T387Y, G408R, A438R, T492Y | OP947114 |
|  |  | 1 | L40508 | 124–491 | T135C, T183Y, C255Y, C273Y, C345Y, T366Y, C372Y, T387Y | OP947115 |
|  |  | 1 | L40508 | 76–489 | T135Y, T183Y, C255T, C273Y, T366Y, T387C, G408R, C411Y, A438R | OP947116 |
|  |  | 1 | L40508 | 76–444 | T135Y, G225R, C255T, C273Y, T366Y, T387C, G408R, C411Y, A438G | OP947117 |
