# Supplementary material for: High Diversity of Giardia duodenalis Assemblages and Sub-Assemblages in Asymptomatic School Children in Ibadan, Nigeria
Source: Trop Med Infect Dis. 2023 Feb 28;8(3):152. doi: 10.3390/tropicalmed8030152 (PMC10051407; doi:10.3390/tropicalmed8030152)
Supplement: Supplementary file 1 [file tropicalmed-08-00152-s001.zip › Table S6 Tijani et al TMID_2022.docx]

**Table S6.** Frequency and molecular diversity of *G. duodenalis* identified at the *bg* locus in the schoolchildren population investigated in the present study. GenBank accession numbers are provided.

| **Assemblage** | **Sub-assemblage** | **No. isolates** | **Reference sequence** | **Stretch** | **Single nucleotide polymorphisms** | **GenBank ID** |
| --- | --- | --- | --- | --- | --- | --- |
| A | AII | 7 | AY072723 | 93–718 | None | OP947118 |
|  |  | 1 | AY072723 | 98–727 | C720T | OP947119 |
|  | AIII | 4 | AY072724 | 93–719 | None | OP947120 |
| B | – | 2 | AY072727 | 97–719 | None | OP947121 |
|  |  | 1 | AY072727 | 93–605 | C111Y, C162Y, C165Y, A183R, C249Y, C309Y, C321Y, G327R, C450Y, C483Y, C516Y, G531R, C564Y | OP947122 |
|  |  | 1 | AY072727 | 98–631 | C130Y, A183G, G261A, C309T, T519C, C579T | OP947123 |
|  |  | 1 | AY072727 | 93–719 | C165T, T519C | OP947124 |
|  |  | 1 | AY072727 | 102–583 | 183G, C309T, G429A, A432G, C441T, T519C | OP947125 |
|  |  | 1 | AY072727 | 93–604 | A183G, C309T, T519C, C564T | OP947126 |
|  |  | 1 | AY072727 | 98–719 | A183R, C309T, T519C, C564T, C609T, G639A | OP947127 |
|  |  | 1 | AY072727 | 98–707 | C309Y, C435Y | OP947128 |
|  |  | 1 | AY072727 | 93–729 | T471Y | OP947129 |
|  |  | 1 | AY072727 | 98–724 | T471Y, C720T | OP947130 |
