# Supplementary material for: High Diversity of Giardia duodenalis Assemblages and Sub-Assemblages in Asymptomatic School Children in Ibadan, Nigeria
Source: Trop Med Infect Dis. 2023 Feb 28;8(3):152. doi: 10.3390/tropicalmed8030152 (PMC10051407; doi:10.3390/tropicalmed8030152)
Supplement: Supplementary file 1 [file tropicalmed-08-00152-s001.zip › Table S7 Tijani et al TMID_2022.docx]

**Table S7.** Frequency and molecular diversity of *G. duodenalis* identified at the *tpi* locus in the schoolchildren population investigated in the present study. GenBank accession numbers are provided.

| **Assemblage** | **Sub-assemblage** | **No. isolates** | **Reference sequence** | **Stretch** | **Single nucleotide polymorphisms** | **GenBank ID** |
| --- | --- | --- | --- | --- | --- | --- |
| A | AII | 5 | U57897 | 292–805 | None | OP947131 |
|  | BIII | 1 | AF069561 | 1–456 | C34Y | OP947132 |
|  |  | 1 | AF069561 | 1–456 | G105A, A135G | OP947133 |
|  |  | 1 | AF069561 | 1–441 | G105R, C255Y | OP947134 |
|  |  | 1 | AF069561 | 1–456 | G108Y, G198R, G207R, G402R | OP947135 |
|  |  | 1 | AF069561 | 1–456 | G198R, G207R, G402R, G436R | OP947136 |
|  |  | 1 | AF069561 | 1–456 | G199R | OP947137 |
|  | BIII/BIV | 1 | AF069560 | 1–404 | T57Y, T131Y, T134Y, A176G, A395G | OP947138 |
