# Supplementary material for: High Diversity of Giardia duodenalis Assemblages and Sub-Assemblages in Asymptomatic School Children in Ibadan, Nigeria
Source: Trop Med Infect Dis. 2023 Feb 28;8(3):152. doi: 10.3390/tropicalmed8030152 (PMC10051407; doi:10.3390/tropicalmed8030152)
Supplement: Supplementary file 1 [file tropicalmed-08-00152-s001.zip › Table S8 Tijani et al TMID_2022.docx]

**Table S8**. Input predictor values for The TwoStep Cluster Analysis, with positivity to *Giardia duodenalis* as evaluation variable.

| **Variable** | **Predictor values** | **Most frequent category (%) Cluster 1** | **Most frequent category (%) Cluster 2** |
| --- | --- | --- | --- |
| Sample area | 1.00 | SYI (64.0%) | MO (74.3%) |
| Source of drinking water | 0.42 | Tap (56%) | Well (46.3%) |
| Defecating in the river | 0.40 | Never (45.7%) | Never (99.3%) |
| Handwashing after defecating | 0.24 | Sometimes (76.6%) | Always (51.5%) |
| Defecating in the bush | 0.23 | Never (44.0%) | Never (86.0%) |
| Use of dustbin | 0.14 | Sometimes (38.9%) | Sometimes (70.6%) |
| Treatment of drinking water | 0.14 | None (71.4%) | None (45.6%) |
| Parasite detected | 0.14 | None (84.6%) | None (60.3%) |
| Drinking water storage | 0.12 | Drums (75.4%) | Drums (49.3%) |
| Microscopy results | 0.11 | Negative (84.6%) | Negative (60.3%) |
| Toilet | 0.06 | Pit latrine (56.0%) | Flush toilet (61.0%) |
| Parent occupation | 0.05 | Trader (61.7%) | Trader (87.5%) |
| Covering of drinking water source | 0.05 | Yes (94.9%) | Yes (94.9%) |
| Washing fruit | 0.04 | Sometimes (70.9%) | Sometimes (62.5%) |
| Age | 0.03 | 9.86 | 10.46 |
| Use of Flagyl | 0.03 | Sometimes (90.9%) | Sometimes (82.4%) |
| Flagyl treatment | 0.02 | No (89.7%) | No (93.4%) |
| Age | 0.02 | 10.29 | 9.77 |
| Contact with domestic animals | 0.02 | Yes (75.4%) | Yes (67.6%) |
| Type of domestic animal | 0.01 | Fowl (28.0%) | None (34.6%) |
| Body mass index (kg/m2) | 0.01 | 13.52 | 13.94 |
| Gender | 0.01 | F (51.4%) | F (55.1%) |
